# UNVEILING THE AGING EXPERIENCES OF CHINESE IMMIGRANTS WITH SOCIAL MEDIA POSTS

**DOI:** 10.1093/geroni/igae098.3159

**Published:** 2024-12-31

**Authors:** Xiayu Chen, Wan Wen

**Affiliations:** University of Illinois Urbana-Champaign, Champaign, Illinois, United States; University of Illinois Urbana-Champaign, Champaign, Illinois, United States

## Abstract

Chinese immigrants are among the largest immigrant groups worldwide. As they age, they may encounter a complex array of cultural and social challenges, striving to find a balance between maintaining their cultural values and adapting to the social norms of the migrated countries. With the aim of understanding the community integration and well-being of the aging Chinese immigrants, this study analyzed social media data to uncover topics that reflect their daily living experience. We collected 3,756 posts from January 1, 2013, to January 1, 2024, from the “Evergreen Life” subforum on Wenxuecity.com, a leading Chinese-language BBS forum catering to the overseas Chinese community. Using Latent Dirichlet Allocation topic modeling approach, this study identified five key topics reflecting their aging experiences, including (1) society and life, focusing on navigating societal norms and healthcare systems in migrated countries and life comparisons between China and abroad; (2) overseas cultural experiences, emphasizing personal adaptation stories; (3) family daily life, highlighting intergenerational dynamics and the importance of family support in aging life; (4) physical activities and personal growth, showing efforts towards well-being improvement through exercise and hobbies; (5) religion and spiritual life, demonstrating the role of spirituality in community connection. These findings offer a comprehensive view of the social, cultural, familial, physical, and spiritual aspects of aging among Chinese immigrants, emphasizing the need for culturally sensitive support services to foster a more inclusive environment for this population.

